# Molecular characterization and recent spread of dengue virus type 3 lineage III_B.3.2 in Brazil: in silico analysis of protein motifs and epidemiology

**DOI:** 10.1007/s11262-026-02259-2

**Published:** 2026-07-19

**Authors:** Alicia Pereira de Morais, Rodrigo Cunha Oliveira

**Affiliations:** 1 Universidade Federal da Bahia, Salvador, Bahia Brasil; 2 Present Address: Centro Universitário UniFTC, Salvador, Bahia Brasil; 3 Escola Bahiana de Medicina e Saúde Pública, Salvador, Brasil

**Keywords:** Amino acid motifs, Dengue, Genotype, Brazil

## Abstract

The dengue virus (DENV) belongs to the *Flaviviridae* family and is responsible for a febrile arbovirus disease of global relevance. In Brazil, there has been endemic circulation of the four viral serotypes since the last century, with DENV-3 being frequently associated with more severe clinical conditions and major epidemics. This study aimed to perform the motif characterization of DENV-3 strains circulating in Brazil and its epidemiology. We obtained cases information by serotype from DATASUS/TABNET from 2013 to 2025. Furthermore, 184 complete genomes of Brazilian samples available in the GenBank and GISAID databases were analyzed with the genome reference sequence of DENV-3 (NC_001475.2). The sequences were edited and analyzed with the MAFFT, BioEdit, Nextclade, and GeneDoc software to identify lineages and amino acid motifs and were subsequently submitted to phylogenetic analyses in the IQ-Tree and FigTree programs. The results indicated a historical predominance of serotype one, but with a considerable increase in DENV-2 and DENV-3 cases in 2024 compared to the previous year. By December 2025, DENV-3 cases had already surpassed those of DENV-1 for that year. Nine amino acid motifs were identified throughout the genome, especially sites of N-myristoylation, casein kinase II phosphorylation, protein kinase C phosphorylation, and N-glycosylation. The predominant lineages were 3III_C.2 and 3III_C.2.2 until 2022, when lineage 3III_B.3.2 was introduced in Brazil, being associated with the increase in cases. This genotype presented substitutions and the absence of protein motifs compared to previous lineages, suggesting that such alterations contributed to its replicative success.

**Supplementary Information:**

The online version contains supplementary material available at https://doi.org/10.1007/s11262-026-02259-2.

## Introduction

Dengue is a zoonotic fever caused by the Dengue virus (DENV) and transmitted by the *Aedes aegypti* or *A. albopictus* mosquito [1–3]. DENV is a Flavivirus, arbovirus belonging to the *Flaviviridae* family, with an enveloped viral particle containing a positive-sense single-stranded RNA genome [2, 4]. The viral genome, with approximately eleven thousand nucleotides, classified into four well-known serotypes capable of infecting humans: DENV-1, DENV-2, DENV-3, and DENV-4, with the serotypes 2 and 3 being associated with more severe cases. [4].

Its genetic material consists of non-coding 5′ and 3′ regions and a single open reading frame (ORF) that encodes a polyprotein, which is later translated into three structural proteins (C, M, and E) and seven non-structural proteins (NS1, NS2A, NS2B, NS3, NS4A, NS4B, and NS5) [5]. Among the structural proteins, the C (capsid) protein is responsible for the assembly and exit of the virus from the infected cell in the viral cycle [6–8]. The membrane (M) provides robustness to the viral structure and stabilizes the fusion peptide of the E protein [5, 9, 10]. The E glycoprotein is in the envelope and stabilizes the protein structure for the binding process to receptors for the virus to enter the cell [11]. The envelope protein binds to the mannose receptor and the CD14 complex in immune system cells, as well as to heparan sulfate in epithelial cells to invade the target cell [12, 13]. The non-structural (NS) proteins have regulatory functions in the viral replication process [14].

Dengue has been a health problem in Southeast Asia and African countries since before the twentieth century [15]. It is hypothesized that the virus was introduced to Brazil during the colonial period, through the trafficking of enslaved people from Africa [16]. In Brazil, the first epidemics with serotype identification were documented in the 1980s, when the concurrent circulation of serotypes 1 and 4 was observed in the state of Roraima [16]. During that same decade, the virus spread to the northeastern and southeastern regions of the country and subsequently expanded to other regions of Brazil. Beginning in the 2000s, infections by serotypes 2 and 3 began to be identified, thereby contributing to an increase in the epidemiological complexity of the disease [4, 17].

The wide dissemination of dengue in the national territory is associated with the climatic conditions in tropical and subtropical regions, characterized by elevated temperatures and high humidity, factors that contribute to the proliferation of the *A. aegypti* vector [18]. Furthermore, the process of disorderly urbanization and the intensification of globalization, especially from the 1980s onward, contributed to the increase in the occurrence of epidemics, alerting the population and public agencies, particularly in Latin America and Brazil [17].

Recent epidemiological studies reinforce this scenario. Menezes et al. (2021) demonstrated that, in the period from 2010 to 2019, most confirmed dengue cases registered in DATASUS occurred in urban areas [19]. Similarly, Silva et al. (2024), when analyzing reported cases between 2014 and 2024, identified the Southeast region as the most affected, followed by the Northeast and Midwest regions, with significant peaks in the years 2015, 2016, and 2019 [20]. Moreover, data presented by Castro et al. (2024) indicate that serotype 1 were the most prevalent among the cases registered in Brazil in 2023 [21].

In February 2025, the Pan American Health Organization (PAHO) issued an epidemiological alert regarding the circulation of dengue virus serotype 3 (DENV-3) in the Americas. They highlighted widespread population susceptibility due to a lack of prior exposure to a serotype considered to cause more severe disease [22]. DENV-3 outbreaks were first recorded in the states of Rio de Janeiro and São Paulo in the early 2000s, following the introduction of the virus via travelers from the Caribbean region [23]. These initial outbreaks remained contained, leading to a subsequent decline in DENV-3 incidence and a resurgence of serotypes 1 and 2 [24, 25]. However, in 2024, the serotype 3 re-emerged, particularly in the Southeast region, associated with more severe cases of the disease [25, 26].

Currently, the incidence of the disease in Brazil is associated with the hot and humid months [17]. Disease control is achieved by attempting to contain mosquito breeding sites and increasing dengue vaccination coverage, such as with the tetravalent vaccines Dengvaxia® (Sanofi Pasteur), available in the private health network since 2015, and Qdenga® (Takeda Pharmaceutical Company), available in the Unified Health System of Brazil since 2023 [27]. High case rates are favored by human displacement between cities, as well as climate changes and population growth, contributing to dengue continuing to be a public health problem [28]. Therefore, this study aims to perform the molecular characterization of DENV-3 strains in Brazil over the years and to evaluate and identify mutations in the structural motifs in the amino acid sequences of Brazilian samples.

## Materials and methods

For the analysis of epidemiological data and the number of confirmed cases, the Brazilian government platform DATASUS/TABNET (https://datasus.saude.gov.br/informacoes-de-saude-tabnet/) was used [29]. Data collection comprised the total number of reported and confirmed dengue cases across Brazilian states, from 2013 to December 2025. Cases were defined based on laboratory confirmation or clinical-epidemiological diagnosis during medical consultations. Concurrently, data regarding the absolute number of confirmed cases were categorized by viral serotype for the identical period. A limitation of this study is that comprehensive data stratified by serotype were only available from 2013 onward and the genomic sequencing data has only increased over the last 6 years, missing previous serotype information.

DENV-3 nucleotide sequences were retrieved from the GISAID database [30, 31] using the following inclusion filters: “complete coding DNA sequences (Cpl. CDS),” “high coverage,” “complete collection date,” and “location: Brazil.” Other exclusion criteria were sequences missing a complete gene or having less than 8000 nucleotides in length. This search encompassed all records available on the platform up to April 1, 2025, yielding 96 sequences (*N* = 96). Additionally, complete coding sequences were compiled from the National Center for Biotechnology Information (NCBI) database [32], resulting in a final dataset of 184 sequences.

Sequences were aligned on the MAFFT online server using the NCBI DENV-3 complete genome reference sequence (NC_001475.2; final *N* = 185) [32, 33]. To correct the reading frame, genomes were edited in BioEdit v7.2.5 through translation alignment and frameshift correction [34]. Protein motifs and conserved regions were then identified in GeneDoc v2.7.000 based on PROSITE patterns. Lineage classification and quality score of the genomes were performed using Nextclade v3.18.1 [35–37].

Subsequently, the sequences were edited again to remove initial and final fragments with no coding regions and got submitted to Maximum Likelihood phylogeny by the IQ-Tree online server (http://www.iqtree.org) with ModelFinder [38, 39]. The automatically selected substitution model was TIM2 + F + I + G4 and branch support using both the SH-like approximate likelihood ratio test (SH-aLRT) and the ultrafast bootstrap approximation (UFBoot) with 1000 replicates [40, 41]. Finally, the tree was observed using the FigTree v1.4.4 software (http://tree.bio.ed.ac.uk/software/figtree/), using the program’s criteria for observation: reroot tree with NC_001475.2, showing SH-aLRT (Approximate Likelihood Ratio Test) and Bootstrap (BS) values, and using the increasing viewing mode [42].

## Results

### Number of cases

According to epidemiological data retrieved from DATASUS, a total of 15,970,928 confirmed dengue cases were recorded in Brazil between 2014 and December 2025. Case confirmation relied on either laboratory tests or the patient’s clinical-epidemiological aspect, with the peak of dengue cases registered in the year 2024 (5,988,245). Furthermore, the Southeast region exhibited the highest concentration of cases, followed by the South, Midwest, Northeast, and North regions. The primary affected group consisted of individuals aged 20–39, followed by the 40–59 and 15–19 age groups.

Among identified serotypes, the total value found available on the platform was 343,139 cases with DENV-1 predominant infections, presenting 215,066, 62.67% of the total. This was followed by DENV-2 (34.27%), DENV-3 (2.56%), and DENV-4 (0.50%). In 2024, a considerable increase was observed in the number of registered DENV-3 infections (1,792 cases) compared to 2023 (106 cases), attributed to the intensification of the circulation of this serotype in national territory. By December 2025, DENV-3 cases had escalated further to 6766 registered infections, exceeding the previous year’s total, as shown in Table 1.

**Table 1:** Probable cases per year and serotype of the dengue virus

Year	DENV-1	DENV-2	DENV-3	DENV-4
2014	2321	30	12	607
2015	5164	67	23	356
2016	3770	130	40	103
2017	273	204	9	51
2018	443	693	3	11
2019	4167	7995	10	397
2020	2035	5082	3	38
2021	2718	1557	3	8
2022	18,093	2462	4	19
2023	40,719	6003	106	17
2024	129,508	57,667	1792	52
2025	5854	35,714	6766	40

Source: Ministry of Health of Brazil/SVSA—Notifiable Diseases Information System —Sinan Net–2025 data updated on 02/02/2026 [29]

## Analysis of the DENV-3 dataset

An exploratory analysis of the 184 DENV-3 sequences based on the reference genome identified nine distinct protein motifs across the multiple sequence alignment (listed in Table 2). Among these, four correspond to protein kinase phosphorylation sites: Casein kinase II (CK2) motifs occurred 54 times (including 19 within NS5); Protein kinase C (PKC) sites were detected 39 times (with 9 located in NS3); cAMP-dependent protein kinase (PKA) sites appeared 10 times, three of which were mapped to the anchored capsid (ancC) protein; and tyrosine kinase phosphorylation sites were observed only six times.

**Table 2:** Protein motifs identified across DENV-3 genes and their frequencies. Mapping was performed using GeneDoc, based on the NC_001475.2 reference sequence and PROSITE consensus patterns

	ancC (95..436); C (95..394)	prM (437..934)	pr (437..709)	M (710..934)	E (935..2413)	NS1 (2414..3469)	NS2A (3470..4123)	NS2B (4124..4513)	NS3 (4514..6370)	NS4A (6371..6751)	2 K (6752..6820)	NS4B (6821..7564)	NS5 (7565..10264)	TOTAL
cAMP- and cGMP- dependent protein kinase phosphorylation site	3	0	0	0	2	0	2	0	0	0	0	1	2	10
Casein kinase II phosphorylation site	0	1		0	8	4	4	3	9	0	1	5	19	54
Protein kinase C phosphorylation site	0	4			5	6	5	2	9	1	0	0	7	39
ATP/GTP-binding site motif A (P-loop)	0	1	0	0	0	0	0	0	0	0	0	0	0	1
N- myristoylation site	1	2			13	7	5	2	13	1	0	3	8	55
N- Glycosylation site	0	1		0	3	1	1	0	1	0	0	2	3	12
Tyrosine kinase phosphorylation site	0	0	0	0	0	1	0	0	4	0	0	0	1	6
RGD cell-attachment sequence	0	0	0	0	0	0	0	0	1	0	0	0	0	1
Amidation site	0	0	0	0	0	1	1	0	3	0	0	1	3	9

Additionally, *N*-myristoylation sites were highly prevalent, appearing 55 times across viral polyprotein, with a notable concentration in both the envelope (E) and NS3 proteins (13 sites each). Finally, 12 asparagine-linked glycosylation (N-glycosylation) sites were identified, distributed across E (3), NS5 (3), NS4B (2), and one site each within prM/pr, NS1, NS2A, and NS3.

Lineage assignment via Nextclade revealed that the reference sequence belongs to lineage 3III_A, which may account for the variations in motif presence or absence observed at the specific amino acid positions detailed in Table 3. Within the analyzed dataset, lineage 3III_B.3.2 was the most prevalent (*N* = 95), followed by 3III_C.2 (*N* = 58), 3III_C.2.2 (*N* = 28), and a single sequence from 2003 classified as lineage 3III_C.1. The accession numbers for all sequences are provided in the supplementary material 3. Most samples collected prior to 2014 belonged to lineage 3III_C, except for those classified under genotype 3V. Overall, the sequences exhibited high conservation, sharing the majority of the identified protein motifs, with the most pronounced variations summarized in Table 3.

**Table 3:** Protein motif variations identified within the sequence alignment relative to the DENV-3 reference genome NC_001475.2

Amino Acid Position	Reference motif	Variant motif	Sequences (GISAID/GenBank ID)
951	No motif at the position	PKC phosphorylation site	EPI_ISL 19704148; EPI_ISL 19750071; EPI_ISL 19750068; EPI_ISL 19750073; EPI_ISL 19750074; EPI_ISL 19752058; EPI_ISL 19750072; GB GU131874.1
1206	CK2 phosphorylation site	Stop códon*	**EPI_ISL_19179567; **EPI_ISL_19179568; **EPI_ISL_19179569
1433	No motif at the position	N-glycosylation site	EPI_ISL_19218804; EPI_ISL_19609376
1861	No motif at the position	PKC phosphorylation site	EPI_ISL_19167318; EPI_ISL_19167320; EPI_ISL_19167323; EPI_ISL_19167319; EPI_ISL_19210548; EPI_ISL_19167322; EPI_ISL_19167321; EPI_ISL_19210549; EPI_ISL_19210546; EPI_ISL_19210547; EPI_ISL_19459684; EPI_ISL_19459672; EPI_ISL_19218804; EPI_ISL_19609376; EPI_ISL_19459690; EPI_ISL_18953953; EPI_ISL_18953960; EPI_ISL_19076632; EPI_ISL_19076634; EPI_ISL_19076640; EPI_ISL_19076633; EPI_ISL_19076637; EPI_ISL_19076642; EPI_ISL_19076631; EPI_ISL_19076641; GU131877.1
2157	CK2 phosphorylation site	Absent	All
2710	CK2 phosphorylation site	Absent	All genomes collected after 2014, except: EPI_ISL_19076641
2890–2891	N-myristoylation	PKC phosphorylation site	All, except: EPI_ISL_19668567; EPI_ISL_19687445; EPI_ISL_19609377; EPI_ISL_18977784; EPI_ISL_19687443; EPI_ISL_19668566; EPI_ISL_19687451

^*^Stop codons at these positions (end of the protein NS2A) result in truncation

^**^These sequences were flagged with a ‘mediocre’ quality status by Nextclade (QC score ≈ 61.1), strongly indicating a sequencing artifact

Notably, premature stop codons were identified in three genomes at amino acid position 1206: EPI_ISL_19179567, EPI_ISL_19179568, and EPI_ISL_19179569. The GISAID annotations on these sequences mutation, located within the NS2A protein, “results in a 77.5% truncation of the NS2A protein,” resulting in a non-functional virus. Also, the lineages assignment on Nextclade also reveals the sequence’s quality and the closest it gets to 0, means a better sequencing quality. These three sequences were qualified as “mediocre” (qc.overallStatus), with a quality score (qc.overallScore) of approximately 61.1, suggesting a sequencing or assembly artifact and not a biological variant.

The absence of motifs at position 2710aa in the NS5 gene in the most recent sequences may be a result of the new circulating genotype since 2022. The 3III_B lineage from the dataset presents this amino acid alteration, suggesting the replicative success of this alteration. Only one 2024 sample from Minas Gerais also does not present this motif; however, it clusters with the samples of the same 3III_B.3.2 lineage. The motif found at position 1433 may be related to host particularities or local circulation.

**Fig. 1:**
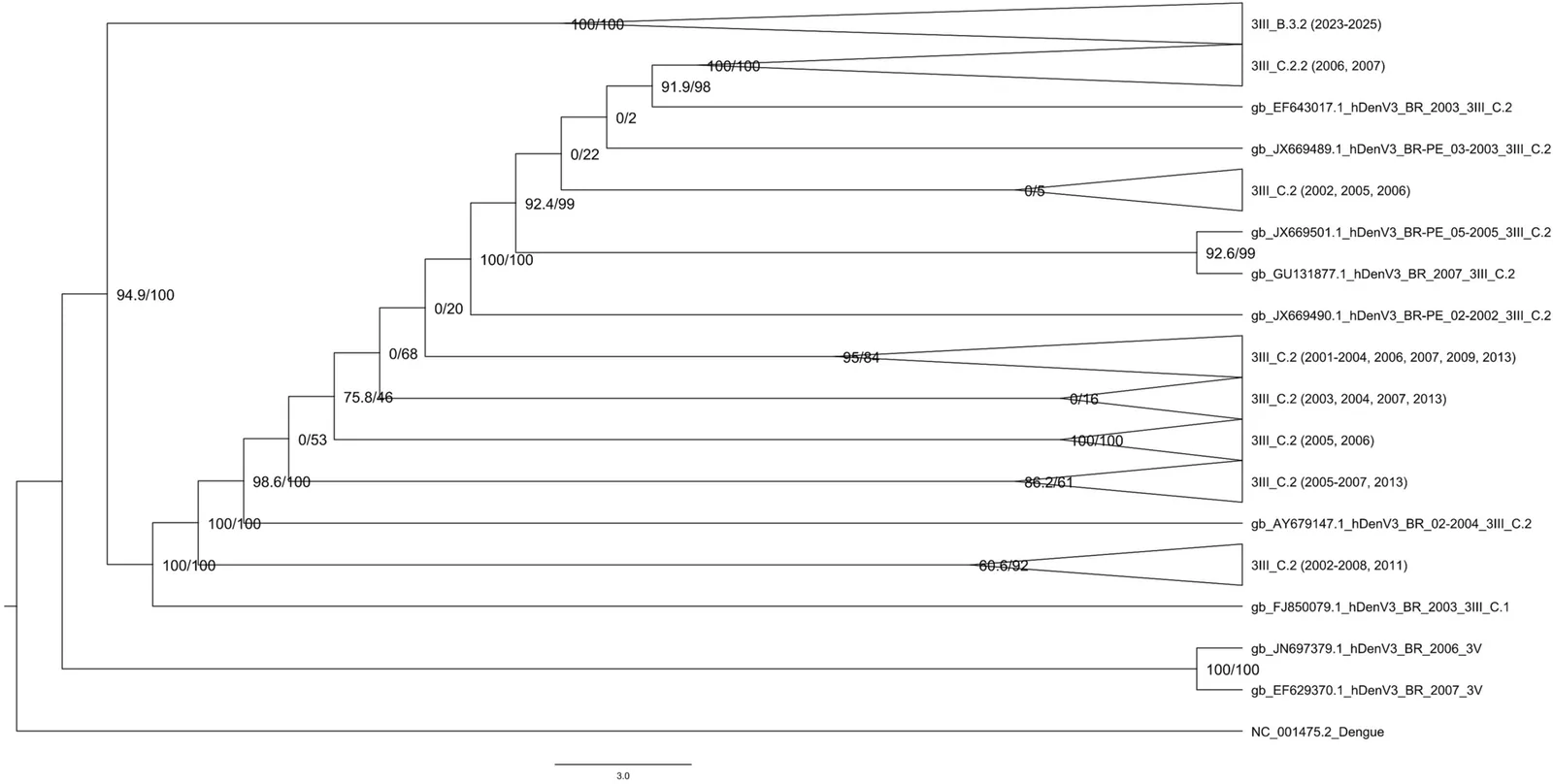
Phylogenetic tree of DENV-3 sequences inferred using the maximum likelihood method. Clades collapsed by lineage and sequence years. Branch support values are shown at the nodes and correspond to SH-aLRT and ultrafast bootstrap support (SH-aLRT/UFboot) calculated from 1000 replicates each. The dataset comprised 184 sequences and the reference NC_001475.2

The maximum likelihood phylogenetic tree rooted with the reference sequence (Fig. 1 and Supplementary material) reveals two major distinct clades, showing temporal evolution of the circulating lineages. The basal clade presents good statistical support from the SH-aLRT and Bootstrap values (94.9/100), grouping the 3III_C.1 (*N* = 1), 3III_C.2, and 3III_C.2.2 lineages with genomes collected mostly between 2001 and 2013 across different Brazilian regions.

Furthermore, two sequences from 2006 and 2007 of genotype 3V were found. They are located on a basal and external branch of the tree ungrouped in relation to the rest, confirming the genetic variability among the genotypes. They present similar protein characteristics to the other sequences. The differences are absence of the N-myristoylation motif at amino acid positions 691, 1144, and 2072; presence of a casein kinase II phosphorylation site at 944; presence of a creatine phosphokinase phosphorylation site at aa positions 1270 and 3105; and absence of N-glycosylation at 2810aa.

In contrast, the upper clade comprised the recent genomes from the lineage 3III_B.3.2. This cluster represents the sequences circulating during the period from 2023 to 2025 from all Brazil regions. Moreover, the branch support (100/100—SH-aLRT/UFboot) may suggest an evolutionary and dispersion success of the lineage in the country.

## Discussion

In the alignment, phosphorylation motifs appear more frequently in DENV-3 non-structural proteins, which may contribute to viral biosynthesis and immune evasion. Protein kinases catalyze the phosphorylation of proteins, resulting in cellular stimuli and responses [43]. NS5 is known to be phosphorylated by casein kinase G and protein kinase C (PKC), and the inhibition of PKC in this protein increases viral replication [44–46]. In our dataset, PKC phosphorylation sites were more frequently in the NS3 and NS1 proteins, which regulate viral replication and polyprotein cleavage. These findings suggest that the identified PKC phosphorylation sites may participate in regulatory signaling pathways associated with the viral replication complex.

Studies with DENV and other viruses demonstrate that activation and signaling pathways mediated by cyclic AMP-dependent protein kinase (PKA) regulate and facilitate viral replication [47–52]. Out of the 10 PKA phosphorylation motifs identified across the entire genome, three were mapped within the anchored capsid (ancC) protein, which may suggest an improvement in the signaling and cleavage of the C protein, consequently enhancing viral assembly.

Guerra and Issinger (1999) demonstrated that casein kinase 2 (CK2) participates in the biosynthesis of viruses such as those from the Herpes, HIV, HPV, and Influenza [53]. In our alignment, the protein with the highest number of CK2 phosphorylation sites is NS5. However, studies have already shown that NS5 presents a cytoplasmic isoform (which interacts with NS3) and a nuclear one, the most usual form in DENV-3 [54–56]. In DENV-2, CK2 phosphorylation on threonine 395 of NS5 favors a reduction in the transport of the protein to the nucleus, which may hinder replication [57]. For the Hepatitis C virus (also a flavivirus), this phosphorylation on the NS5A protein is essential for viral replication [58]. With nineteen sites on NS5, nine on NS3, and eight on the E protein, it is hypothesized that these motifs may exert a complex regulatory influence on the replicative success of DENV-3.

In silico tests demonstrated indicative results that *N*-myristoylation sites are abundant in all four DENV serotypes, with emphasis on the E, NS5, and NS3 proteins, which corroborates the findings in our analyses [59]. Consistently, our analysis revealed an elevated frequency of these motifs within these exact DENV-3 proteins. It could potentially contribute to greater efficacy in the virus’s entry process into the target cell and potentially stabilize replication complexes from the synthesis of fatty acids at the replicative sites, as occurs in DENV-2 [60].

Within the dataset analyzed, 12 asparagine-linked glycosylation sites were mapped. The *N*-glycosylation of viral proteins is predicted to improve the binding between the virus and the cell and the replicative process [61]. Previous works have demonstrated that the E protein normally presents two glycosylation sites and, after being glycosylated, interacts with prM for its folding, resulting in improved virulence and infectivity [62–64]. Works with DENV-2 demo nstrated that *N*-glycosylation on NS1 stabilizes the protein and promotes the exit of the virus from the cell [65, 66].

According to Heaton et al. (2010) and Bagdonaite and Wandall (2018), the presence of motifs such as *N*-myristoylation and *N*-glycosylation improves the binding between the virus and the host cell [60, 61]. In contrast, the absence of these motifs can hinder the virus’s binding and entry process into the cell [60, 61]. The presence of these motifs in our DENV-3 alignment suggests that these predicted *N*-glycosylation profiles participate in viral maturation and host cell exit pathways.

The 3III genotype has been responsible for the highest number of cases in Brazil since the first epidemics described in the early 2000 s [24]. It is believed that this genotype emerged in the 1960s in the Indian region, spread to Sri Lanka and Africa in the 1980s, and then reached Latin America in the 1990s, causing moderate or severe cases of dengue [67]. Historical reconstructions indicate that genotype 3III was introduced to Brazil from the Caribbean islands, Colombia, and Venezuela, undergoing regional diversification and forming subclades [68].

In our dataset, lineages 3III_C.2, 3III_C.2.2, and 3III_B.3.2 are predominant. Lineage 3III_B.3.2 appears from 2023 to 2025, corroborating the work of Pereira et al. (2025), which shows the introduction of this lineage in Brazil in 2022 [69]. Because DENV-3 exhibited low circulation in the country for more than fifteen years, the result was an immunological gap in the population, as alerted by PAHO [22]. The introduction of 3III_B.3.2 into this susceptible immunological landscape and the rapid dissemination, evidenced by our tree topology and epidemiological data, reflects the virus’s evolutional trajectory and may explain its evolutionary success and its potential for epidemics in Brazil and neighboring countries.

The taxonomic definition of the 3V genotype was controversial among researchers. Lanciotti and colleagues (1994) classified samples of this genotype as a subgroup of 3I [70]. However, Nogueira et al. (2008) and Araújo et al. (2009) found Brazilian sequences of this lineage that cluster with samples from the Asian continent from the years 1956, 1973, and 1980 that did not closely approach other genotypes, and the classification was recognized [71, 72]. This cluster remained phylogenetically distinct and did not group with other established genotypes, corroborating to its status as an independent genotype.

## Conclusion

In this work, the epidemiological evolution of DENV-3 in Brazil and the structural motifs found in sequences isolated in the country were observed. The emergence of the 3III_B.3.2 lineage in 2022 led to a rise in DENV-3 cases, which raised concern about an epidemic. This lineage presents similar characteristics to the lineage that circulated in the country previously (3III_C.2 and 3III_C.2.2), having few differences between motifs, except at positions 1861aa and 2710aa, which may suggest that these changes favored the proliferation of the virus in Brazil.

Nonetheless, our study is limited to the samples deposited in public databases, considering the quality of these sequences and the tools used in this work. Our genomic surveillance relies entirely on sequences deposited in public databases. This dependency may introduce geographical and temporal sampling biases, as sequencing efforts vary across different regions, potentially underrepresenting certain areas or early phases of the outbreak. Also, this study has a predictive characterization, using exclusively computational analysis. Furthermore, in vivo studies should be conducted to understand the particularities of each serotype and genotype of the dengue virus and to analyze the impact of the protein motifs and structures of the circulating genotypes. In this way, it will be possible to predict and prevent new epidemics of DENV-3 in Brazil 

## Supplementary Information

Below is the link to the electronic supplementary material.

Supplementary file1 (PDF 111 KB)

## Data Availability

All epidemiological data on Dengue fever used in this study are available on the DATASUS/TABNET website (https://datasus.saude.gov.br/informacoes-de-saude-tabnet/). The genomic sequences were obtained from the National Center for Biotechnology Information (https://www.ncbi.nlm.nih.gov/nuccore/?term =) and GISAID (https://gisaid.org/collaborations/arbovirus-researchers-unite/); all accession numbers are provided in the Supplementary Information.
