# Supplementary material for: Molecular characterization and recent spread of dengue virus type 3 lineage III_B.3.2 in Brazil: in silico analysis of protein motifs and epidemiology
Source: Virus Genes. 2026 Jul 19;62(5):587–95. doi: 10.1007/s11262-026-02259-2 (PMC13615040; doi:10.1007/s11262-026-02259-2)
Supplement: Supplementary file 1 [file 11262_2026_2259_MOESM1_ESM.pdf]

SUPPLEMENTARY INFORMATION

Molecular characterization and recent spread of Dengue Virus Type 3 Lineage III_B.3.2 in Brazil: *in silico* analysis of protein motifs and epidemiology

Alicia Pereira de Morais[1] e Rodrigo Cunha Oliveira

[1]Universidade Federal da Bahia, Salvador, Bahia, Brasil – aliciamorais100@outlook.com – ORCID 0009-0002-8683-5924

[2]UNIDOMPEDRO and UniFTC, Salvador, Bahia Brasil. ORCID 0000-0001-7324-8793

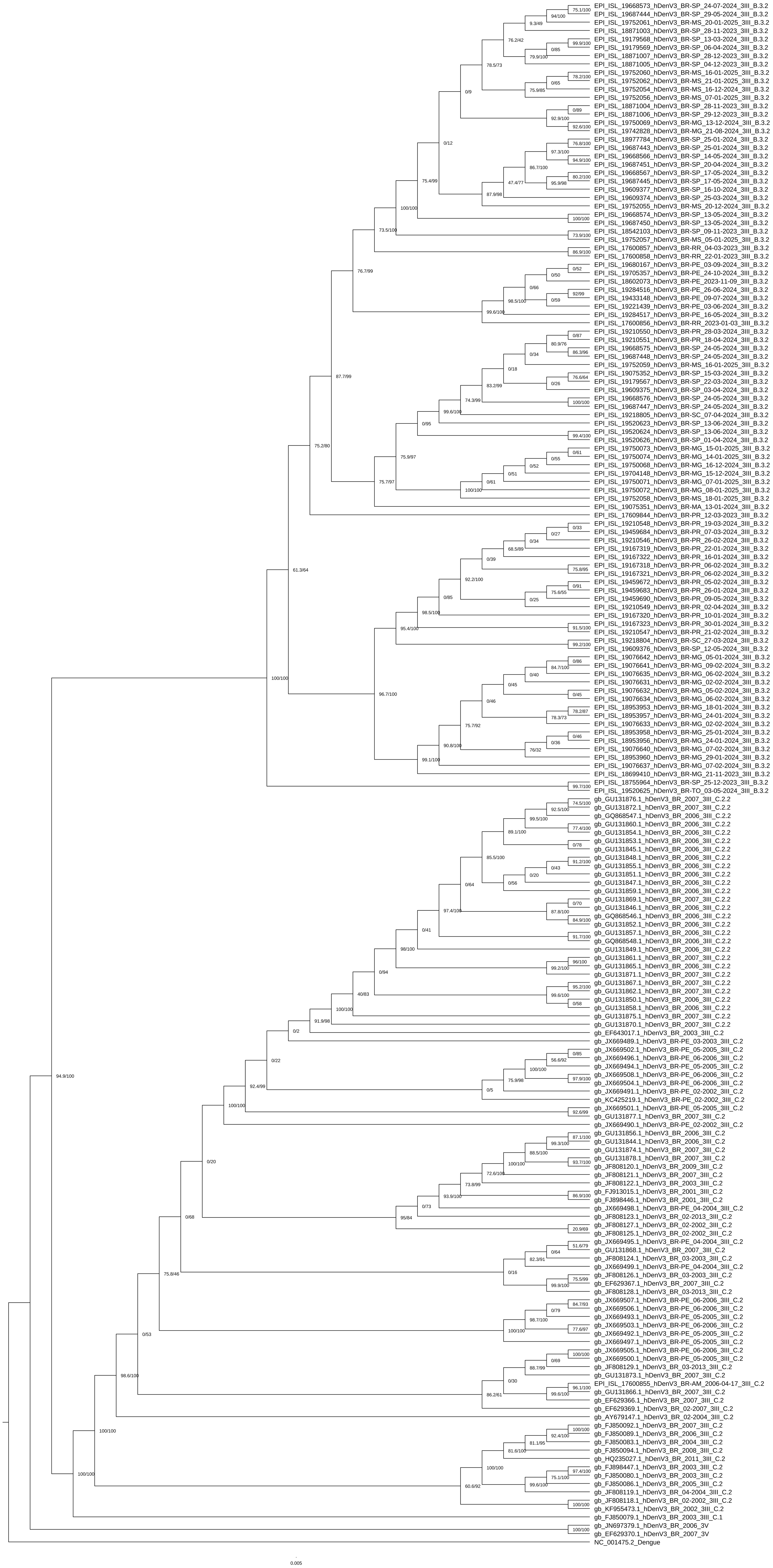
EPI_ISL_19668573_hDenV3_BR-SP_24-07-2024_3III_B.3.2
EPI_ISL_19687444_hDenV3_BR-SP_29-05-2024_3III_B.3.2
EPI_ISL_19752061_hDenV3_BR-MS_20-01-2025_3III_B.3.2
EPI_ISL_18871003_hDenV3_BR-SP_28-11-2023_3III_B.3.2
EPI_ISL_19179568_hDenV3_BR-SP_13-03-2024_3III_B.3.2
EPI_ISL_19179569_hDenV3_BR-SP_06-04-2024_3III_B.3.2
EPI_ISL_18871007_hDenV3_BR-SP_28-12-2023_3III_B.3.2
EPI_ISL_18871005_hDenV3_BR-SP_04-12-2023_3III_B.3.2
EPI_ISL_19752060_hDenV3_BR-MS_16-01-2025_3III_B.3.2
EPI_ISL_19752062_hDenV3_BR-MS_21-01-2025_3III_B.3.2
EPI_ISL_19752054_hDenV3_BR-MS_16-12-2024_3III_B.3.2
EPI_ISL_19752056_hDenV3_BR-MS_07-01-2025_3III_B.3.2
EPI_ISL_18871004_hDenV3_BR-SP_28-11-2023_3III_B.3.2
EPI_ISL_18871006_hDenV3_BR-SP_29-12-2023_3III_B.3.2
EPI_ISL_19750069_hDenV3_BR-MG_13-12-2024_3III_B.3.2
EPI_ISL_19742828_hDenV3_BR-MG_21-08-2024_3III_B.3.2
EPI_ISL_18977784_hDenV3_BR-SP_25-01-2024_3III_B.3.2
EPI_ISL_19687443_hDenV3_BR-SP_25-01-2024_3III_B.3.2
EPI_ISL_19668566_hDenV3_BR-SP_14-05-2024_3III_B.3.2
EPI_ISL_19687451_hDenV3_BR-SP_20-04-2024_3III_B.3.2
EPI_ISL_19668567_hDenV3_BR-SP_17-05-2024_3III_B.3.2
EPI_ISL_19687445_hDenV3_BR-SP_17-05-2024_3III_B.3.2
EPI_ISL_19609377_hDenV3_BR-SP_16-10-2024_3III_B.3.2
EPI_ISL_19609374_hDenV3_BR-SP_25-03-2024_3III_B.3.2
EPI_ISL_19752055_hDenV3_BR-MS_20-12-2024_3III_B.3.2
EPI_ISL_19668574_hDenV3_BR-SP_13-05-2024_3III_B.3.2
EPI_ISL_19687450_hDenV3_BR-SP_13-05-2024_3III_B.3.2
EPI_ISL_18542103_hDenV3_BR-SP_09-11-2023_3III_B.3.2
EPI_ISL_19752057_hDenV3_BR-MS_05-01-2025_3III_B.3.2
EPI_ISL_17600857_hDenV3_BR-RR_04-03-2023_3III_B.3.2
EPI_ISL_17600858_hDenV3_BR-RR_22-01-2023_3III_B.3.2
EPI_ISL_19680167_hDenV3_BR-PE_03-09-2024_3III_B.3.2
EPI_ISL_19705357_hDenV3_BR-PE_24-10-2024_3III_B.3.2
EPI_ISL_18602073_hDenV3_BR-PE_2023-11-09_3III_B.3.2
EPI_ISL_19284516_hDenV3_BR-PE_26-06-2024_3III_B.3.2
EPI_ISL_19433148_hDenV3_BR-PE_09-07-2024_3III_B.3.2
EPI_ISL_19221439_hDenV3_BR-PE_03-06-2024_3III_B.3.2
EPI_ISL_19284517_hDenV3_BR-PE_16-05-2024_3III_B.3.2
EPI_ISL_17600856_hDenV3_BR-RR_2023-01-03_3III_B.3.2
EPI_ISL_19210550_hDenV3_BR-PR_28-03-2024_3III_B.3.2
EPI_ISL_19210551_hDenV3_BR-PR_18-04-2024_3III_B.3.2
EPI_ISL_19668575_hDenV3_BR-SP_24-05-2024_3III_B.3.2
EPI_ISL_19687448_hDenV3_BR-SP_24-05-2024_3III_B.3.2
EPI_ISL_19752059_hDenV3_BR-MS_16-01-2025_3III_B.3.2
EPI_ISL_19075352_hDenV3_BR-SP_15-03-2024_3III_B.3.2
EPI_ISL_19179567_hDenV3_BR-SP_22-03-2024_3III_B.3.2
EPI_ISL_19609375_hDenV3_BR-SP_03-04-2024_3III_B.3.2
EPI_ISL_19668576_hDenV3_BR-SP_24-05-2024_3III_B.3.2
EPI_ISL_19687447_hDenV3_BR-SP_24-05-2024_3III_B.3.2
EPI_ISL_19218805_hDenV3_BR-SC_07-04-2024_3III_B.3.2
EPI_ISL_19520623_hDenV3_BR-SP_13-06-2024_3III_B.3.2
EPI_ISL_19520624_hDenV3_BR-SP_13-06-2024_3III_B.3.2
EPI_ISL_19520626_hDenV3_BR-SP_01-04-2024_3III_B.3.2
EPI_ISL_19750073_hDenV3_BR-MG_15-01-2025_3III_B.3.2
EPI_ISL_19750074_hDenV3_BR-MG_14-01-2025_3III_B.3.2
EPI_ISL_19750068_hDenV3_BR-MG_16-12-2024_3III_B.3.2
EPI_ISL_19704148_hDenV3_BR-MG_15-12-2024_3III_B.3.2
EPI_ISL_19750071_hDenV3_BR-MG_07-01-2025_3III_B.3.2
EPI_ISL_19750072_hDenV3_BR-MG_08-01-2025_3III_B.3.2
EPI_ISL_19752058_hDenV3_BR-MS_18-01-2025_3III_B.3.2
EPI_ISL_19075351_hDenV3_BR-MA_13-01-2024_3III_B.3.2
EPI_ISL_17609844_hDenV3_BR-PR_12-03-2023_3III_B.3.2
EPI_ISL_19210548_hDenV3_BR-PR_19-03-2024_3III_B.3.2
EPI_ISL_19459684_hDenV3_BR-PR_07-03-2024_3III_B.3.2
EPI_ISL_19210546_hDenV3_BR-PR_26-02-2024_3III_B.3.2
EPI_ISL_19167319_hDenV3_BR-PR_22-01-2024_3III_B.3.2
EPI_ISL_19167322_hDenV3_BR-PR_16-01-2024_3III_B.3.2
EPI_ISL_19167318_hDenV3_BR-PR_06-02-2024_3III_B.3.2
EPI_ISL_19167321_hDenV3_BR-PR_06-02-2024_3III_B.3.2
EPI_ISL_19459672_hDenV3_BR-PR_05-02-2024_3III_B.3.2
EPI_ISL_19459683_hDenV3_BR-PR_26-01-2024_3III_B.3.2
EPI_ISL_19459690_hDenV3_BR-PR_09-05-2024_3III_B.3.2
EPI_ISL_19210549_hDenV3_BR-PR_02-04-2024_3III_B.3.2
EPI_ISL_19167320_hDenV3_BR-PR_10-01-2024_3III_B.3.2
EPI_ISL_19167323_hDenV3_BR-PR_30-01-2024_3III_B.3.2
EPI_ISL_19210547_hDenV3_BR-PR_21-02-2024_3III_B.3.2
EPI_ISL_19218804_hDenV3_BR-SC_27-03-2024_3III_B.3.2
EPI_ISL_19609376_hDenV3_BR-SP_12-05-2024_3III_B.3.2
EPI_ISL_19076642_hDenV3_BR-MG_05-01-2024_3III_B.3.2
EPI_ISL_19076641_hDenV3_BR-MG_09-02-2024_3III_B.3.2
EPI_ISL_19076635_hDenV3_BR-MG_06-02-2024_3III_B.3.2
EPI_ISL_19076631_hDenV3_BR-MG_02-02-2024_3III_B.3.2
EPI_ISL_19076632_hDenV3_BR-MG_05-02-2024_3III_B.3.2
EPI_ISL_19076634_hDenV3_BR-MG_06-02-2024_3III_B.3.2
EPI_ISL_18953953_hDenV3_BR-MG_18-01-2024_3III_B.3.2
EPI_ISL_18953957_hDenV3_BR-MG_24-01-2024_3III_B.3.2
EPI_ISL_19076633_hDenV3_BR-MG_02-02-2024_3III_B.3.2
EPI_ISL_18953958_hDenV3_BR-MG_25-01-2024_3III_B.3.2
EPI_ISL_18953956_hDenV3_BR-MG_24-01-2024_3III_B.3.2
EPI_ISL_19076640_hDenV3_BR-MG_07-02-2024_3III_B.3.2
EPI_ISL_18953960_hDenV3_BR-MG_29-01-2024_3III_B.3.2
EPI_ISL_19076637_hDenV3_BR-MG_07-02-2024_3III_B.3.2
EPI_ISL_18699410_hDenV3_BR-MG_21-11-2023_3III_B.3.2
EPI_ISL_18755964_hDenV3_BR-SP_25-12-2023_3III_B.3.2
EPI_ISL_19520625_hDenV3_BR-TO_03-05-2024_3III_B.3.2
gb_GU131876.1_hDenV3_BR_2007_3III_C.2.2
gb_GU131872.1_hDenV3_BR_2007_3III_C.2.2
gb_GQ868547.1_hDenV3_BR_2006_3III_C.2.2
gb_GU131860.1_hDenV3_BR_2006_3III_C.2.2
gb_GU131854.1_hDenV3_BR_2006_3III_C.2.2
gb_GU131853.1_hDenV3_BR_2006_3III_C.2.2
gb_GU131845.1_hDenV3_BR_2006_3III_C.2.2
gb_GU131848.1_hDenV3_BR_2006_3III_C.2.2
gb_GU131855.1_hDenV3_BR_2006_3III_C.2.2
gb_GU131851.1_hDenV3_BR_2006_3III_C.2.2
gb_GU131847.1_hDenV3_BR_2006_3III_C.2.2
gb_GU131859.1_hDenV3_BR_2006_3III_C.2.2
gb_GU131869.1_hDenV3_BR_2007_3III_C.2.2
gb_GU131846.1_hDenV3_BR_2006_3III_C.2.2
gb_GQ868546.1_hDenV3_BR_2006_3III_C.2.2
gb_GU131852.1_hDenV3_BR_2006_3III_C.2.2
gb_GU131857.1_hDenV3_BR_2006_3III_C.2.2
gb_GQ868548.1_hDenV3_BR_2006_3III_C.2.2
gb_GU131849.1_hDenV3_BR_2006_3III_C.2.2
gb_GU131861.1_hDenV3_BR_2007_3III_C.2.2
gb_GU131865.1_hDenV3_BR_2006_3III_C.2.2
gb_GU131871.1_hDenV3_BR_2007_3III_C.2.2
gb_GU131867.1_hDenV3_BR_2007_3III_C.2.2
gb_GU131862.1_hDenV3_BR_2007_3III_C.2.2
gb_GU131850.1_hDenV3_BR_2006_3III_C.2.2
gb_GU131858.1_hDenV3_BR_2006_3III_C.2.2
gb_GU131875.1_hDenV3_BR_2007_3III_C.2.2
gb_GU131870.1_hDenV3_BR_2007_3III_C.2.2
gb_EF643017.1_hDenV3_BR_2003_3III_C.2
gb_JX669489.1_hDenV3_BR-PE_03-2003_3III_C.2
gb_JX669502.1_hDenV3_BR-PE_05-2005_3III_C.2
gb_JX669496.1_hDenV3_BR-PE_06-2006_3III_C.2
gb_JX669494.1_hDenV3_BR-PE_05-2005_3III_C.2
gb_JX669508.1_hDenV3_BR-PE_06-2006_3III_C.2
gb_JX669504.1_hDenV3_BR-PE_06-2006_3III_C.2
gb_JX669491.1_hDenV3_BR-PE_02-2002_3III_C.2
gb_KC425219.1_hDenV3_BR-PE_02-2002_3III_C.2
gb_JX669501.1_hDenV3_BR-PE_05-2005_3III_C.2
gb_GU131877.1_hDenV3_BR_2007_3III_C.2
gb_JX669490.1_hDenV3_BR-PE_02-2002_3III_C.2
gb_GU131856.1_hDenV3_BR_2006_3III_C.2
gb_GU131844.1_hDenV3_BR_2006_3III_C.2
gb_GU131874.1_hDenV3_BR_2007_3III_C.2
gb_GU131878.1_hDenV3_BR_2007_3III_C.2
gb_JF808120.1_hDenV3_BR_2009_3III_C.2
gb_JF808121.1_hDenV3_BR_2007_3III_C.2
gb_JF808122.1_hDenV3_BR_2003_3III_C.2
gb_FJ913015.1_hDenV3_BR_2001_3III_C.2
gb_FJ898446.1_hDenV3_BR_2001_3III_C.2
gb_JX669498.1_hDenV3_BR-PE_04-2004_3III_C.2
gb_JF808123.1_hDenV3_BR_02-2013_3III_C.2
gb_JF808127.1_hDenV3_BR_02-2002_3III_C.2
gb_JF808125.1_hDenV3_BR_02-2002_3III_C.2
gb_JX669495.1_hDenV3_BR-PE_04-2004_3III_C.2
gb_GU131868.1_hDenV3_BR_2007_3III_C.2
gb_JF808124.1_hDenV3_BR_03-2003_3III_C.2
gb_JX669499.1_hDenV3_BR-PE_04-2004_3III_C.2
gb_JF808126.1_hDenV3_BR_03-2003_3III_C.2
gb_EF629367.1_hDenV3_BR_2007_3III_C.2
gb_JF808128.1_hDenV3_BR_03-2013_3III_C.2
gb_JX669507.1_hDenV3_BR-PE_06-2006_3III_C.2
gb_JX669506.1_hDenV3_BR-PE_06-2006_3III_C.2
gb_JX669493.1_hDenV3_BR-PE_05-2005_3III_C.2
gb_JX669503.1_hDenV3_BR-PE_06-2006_3III_C.2
gb_JX669492.1_hDenV3_BR-PE_05-2005_3III_C.2
gb_JX669497.1_hDenV3_BR-PE_05-2005_3III_C.2
gb_JX669505.1_hDenV3_BR-PE_06-2006_3III_C.2
gb_JX669500.1_hDenV3_BR-PE_05-2005_3III_C.2
gb_JF808129.1_hDenV3_BR_03-2013_3III_C.2
gb_GU131873.1_hDenV3_BR_2007_3III_C.2
EPI_ISL_17600855_hDenV3_BR-AM_2006-04-17_3III_C.2
gb_GU131866.1_hDenV3_BR_2007_3III_C.2
gb_EF629366.1_hDenV3_BR_2007_3III_C.2
gb_EF629369.1_hDenV3_BR_02-2007_3III_C.2
gb_AY679147.1_hDenV3_BR_02-2004_3III_C.2
gb_FJ850092.1_hDenV3_BR_2007_3III_C.2
gb_FJ850089.1_hDenV3_BR_2006_3III_C.2
gb_FJ850083.1_hDenV3_BR_2004_3III_C.2
gb_FJ850094.1_hDenV3_BR_2008_3III_C.2
gb_HQ235027.1_hDenV3_BR_2011_3III_C.2
gb_FJ898447.1_hDenV3_BR_2003_3III_C.2
gb_FJ850080.1_hDenV3_BR_2003_3III_C.2
gb_FJ850086.1_hDenV3_BR_2005_3III_C.2
gb_JF808119.1_hDenV3_BR_04-2004_3III_C.2
gb_JF808118.1_hDenV3_BR_02-2002_3III_C.2
gb_KF955473.1_hDenV3_BR_2002_3III_C.2
gb_FJ850079.1_hDenV3_BR_2003_3III_C.1
gb_JN697379.1_hDenV3_BR_2006_3V
gb_EF629370.1_hDenV3_BR_2007_3V
NC_001475.2_Dengue
0.005
